# Physiological and Proteomic Analyses of *Saccharum* spp. Grown under Salt Stress

**DOI:** 10.1371/journal.pone.0098463

**Published:** 2014-06-03

**Authors:** Aline Melro Murad, Hugo Bruno Correa Molinari, Beatriz Simas Magalhães, Augusto Cesar Franco, Frederico Scherr Caldeira Takahashi, Nelson Gomes de Oliveira-, Octávio Luiz Franco, Betania Ferraz Quirino

**Affiliations:** 1 Genome Sciences and Biotechnology Program, Universidade Católica de Brasília, Brasília, Distrito Federal, Brazil; 2 Centro de Análises Proteômicas e Bioquímicas, Universidade Católica de Brasília, Brasília, Distrito Federal, Brazil; 3 Embrapa-Agroenergy, Brasília, Distrito Federal, Brazil; 4 Department of Botany, Universidade de Brasília, Brasília, Distrito Federal, Brazil; 5 Department of Ecology, Universidade de Brasília, Brasília, Brazil; Nanjing Agricultural University, China

## Abstract

Sugarcane (*Saccharum* spp.) is the world most productive sugar producing crop, making an understanding of its stress physiology key to increasing both sugar and ethanol production. To understand the behavior and salt tolerance mechanisms of sugarcane, two cultivars commonly used in Brazilian agriculture, RB867515 and RB855536, were submitted to salt stress for 48 days. Physiological parameters including net photosynthesis, water potential, dry root and shoot mass and malondialdehyde (MDA) content of leaves were determined. Control plants of the two cultivars showed similar values for most traits apart from higher root dry mass in RB867515. Both cultivars behaved similarly during salt stress, except for MDA levels for which there was a delay in the response for cultivar RB867515. Analysis of leaf macro- and micronutrients concentrations was performed and the concentration of Mn^2+^ increased on day 48 for both cultivars. In parallel, to observe the effects of salt stress on protein levels in leaves of the RB867515 cultivar, two-dimensional gel electrophoresis followed by MS analysis was performed. Four proteins were differentially expressed between control and salt-treated plants. Fructose 1,6-bisphosphate aldolase was down-regulated, a germin-like protein and glyceraldehyde 3-phosphate dehydrogenase showed increased expression levels under salt stress, and heat-shock protein 70 was expressed only in salt-treated plants. These proteins are involved in energy metabolism and defense-related responses and we suggest that they may be involved in protection mechanisms against salt stress in sugarcane.

## Introduction

Sugarcane (*Saccharum* spp.) is a semi-perennial monocot that can be propagated vegetatively by culms [1], [2]. Its cultivation occurs in more than 80 tropical and subtropical countries [3], [4]. Sugar and bioethanol are the main products obtained from sugarcane and Brazil is one of the largest sugarcane producers of the world [5], [6].

Crop irrigation is essential in arid and semi-arid regions. However, when inappropriately applied, it may result in environmental degradation [7]. Soil salinization has been reported to be one of the causes of soil degradation, menacing productive lands under irrigated agriculture. According to FAO, it is estimated that 34 million hectares (i.e., 11% of the irrigated area) are affected by some level of salinization [8]. The cost of soil salinization to agriculture is estimated to be approximately US$ 12 billion a year. However, this value is expected to increase [9].

High concentrations of salt reduce osmotic potential in soil solution and promote drought stress in plants, which explains the fact that drought and salt stress cause similar symptoms in plants. Salinity imposes diffusive and metabolic limitations to photosynthesis, affects cell growth by restricting water uptake and cell turgor, resulting in increasing accumulation of Na^+^ and Cl^-^ ions inside the cell [10]–[12]. Accumulation of Na^+^ and Cl^-^ ions severely inhibits many photosynthetic enzymes among others and triggers the production of reactive oxygen species (ROS) [13], which can cause plant damage and, in severe cases, death [14]. In an attempt to overcome the toxic effects caused by salinity, plants use various defense mechanisms such as the production of compatible osmolytes (i.e., aminoacids, sugars, and alcohols). These osmolytes balance the osmotic pressure within the cell [15]–[17], thus maintaining root water uptake, plant water balance and photosynthetic activity. They also play a role in membrane and protein protection and scavenging of reactive oxygen species. There is also increased production of certain proteins in response to salt stress, such as superoxide dismutase [10], [18] that eliminates ROS excess, and heat-shock proteins [19] that are responsible for maintaining the correct folding of proteins.

According to the sugarcane cultivar census in Brazil held by the Centro de Tecnologia Canavieira (CTC) [20], the RB (Brazilian Republic) cultivars represent approximately 50% of sugarcane planted in Brazil. Cultivars RB855536 and RB867515 are respectively the second and seventh in farmers' preference, due to traits such as high productivity, erect culms and resistance to diseases [20], [21]. Both cultivars are derived from interspecific hybridizations between *Saccharum officinarum* and *S. spontaneum*. Farmers consider cultivar RB867515 more drought-stress tolerant when compared to cultivar RB855536, although the scant experimental evidence is inconclusive [21]. In fact, water deficit is one of the major factors limiting sugarcane productivity [22]. Given the similarity between drought and salinity responses, we hypothesized that RB867515 would be salt tolerant when compared to RB855536. We assessed the salinity tolerance of the two cultivars by measuring photosynthesis, water potential, macro- and micronutrients and lipid peroxidation of leaves and biomass allocation in response to a long-term period of salt stress (48 days). Additionally, a proteomic approach was used to identify salt stress-induced proteins in cultivar RB867515 that may have biotechnological potential.

## Results

### Photosynthesis and leaf water potential

**Figure 1:**
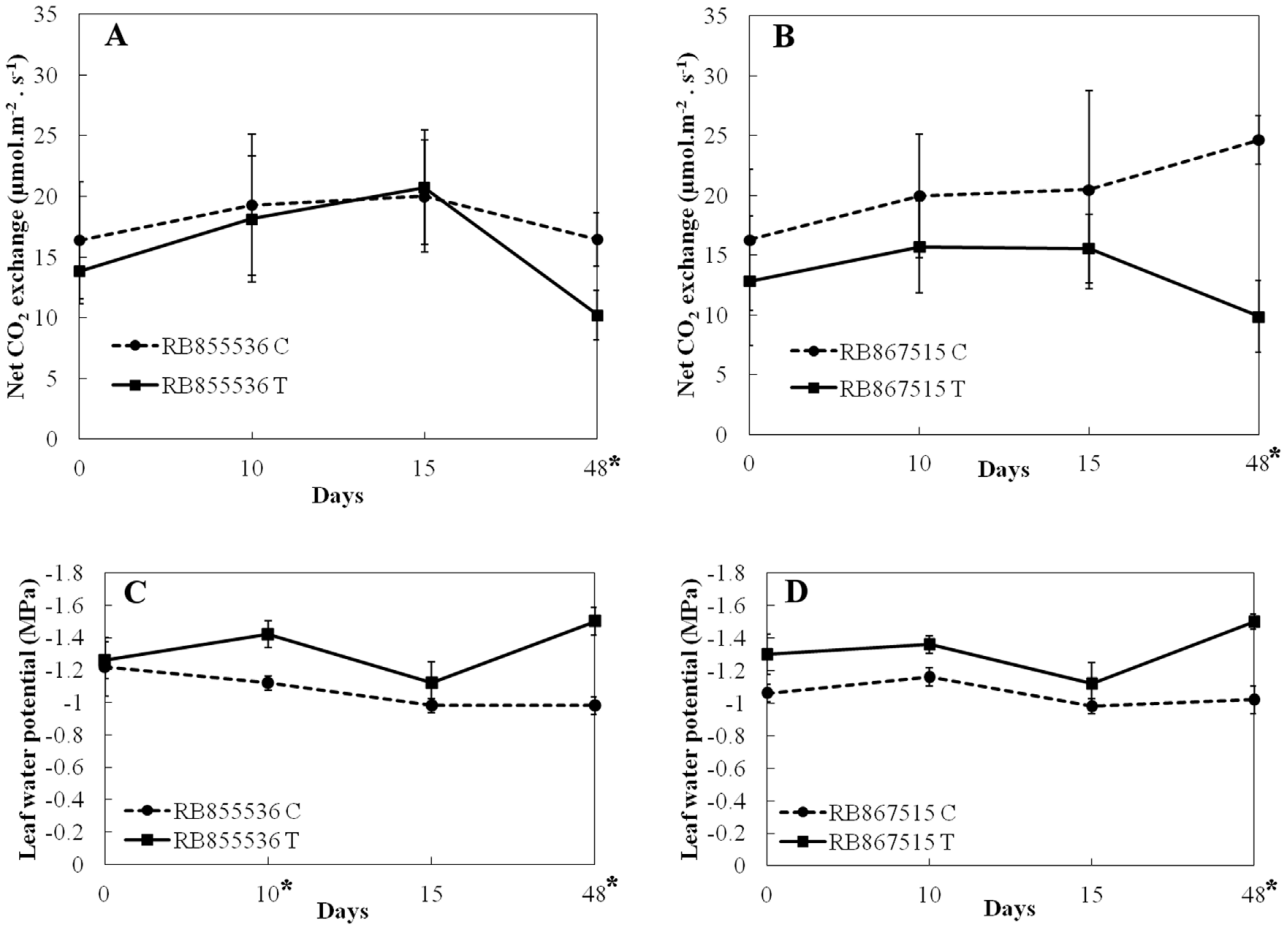
Net CO_2_-exchange (µmol.m^−2^s^−1^) for (A) cultivar RB855536 and (B) cultivar RB867515 over time. Leaf water potential (MPa) in sugarcane leaves during 48 days of salt treatment for (C) cultivar RB855536; and (D) cultivar RB867515. Values are presented as mean ± SD (n = 6). "•" are control plants and "▪" are salt-treated plants. *Significant at p≤0.05.

In both cultivars, RB855536 and RB867515, photosynthetic rates of control and salt-treated plants significantly decreased after 48 days of salt stress (Figure 1A and 1B). However, there were no statistically significant differences between the two cultivars, indicating that the varieties RB855536 and RB867515 behaved similarly with respect to net photosynthesis during salt stress.

Leaf water potential of RB855536 and RB867515 plants subjected to salinity became more negative from day 15 until the end of the experiment (Figure 1C and 1D). At day 48, the water potential of control plants remained at values similar to those of previous timepoints, while salt-treated plants showed a sharp decrease in leaf water potential compared to that of day 15. However, there were no differences in leaf water potential between salt-stressed plants of the two cultivars.

### Biomass allocation and malondialdehyde (MDA) content

**Figure 2:**
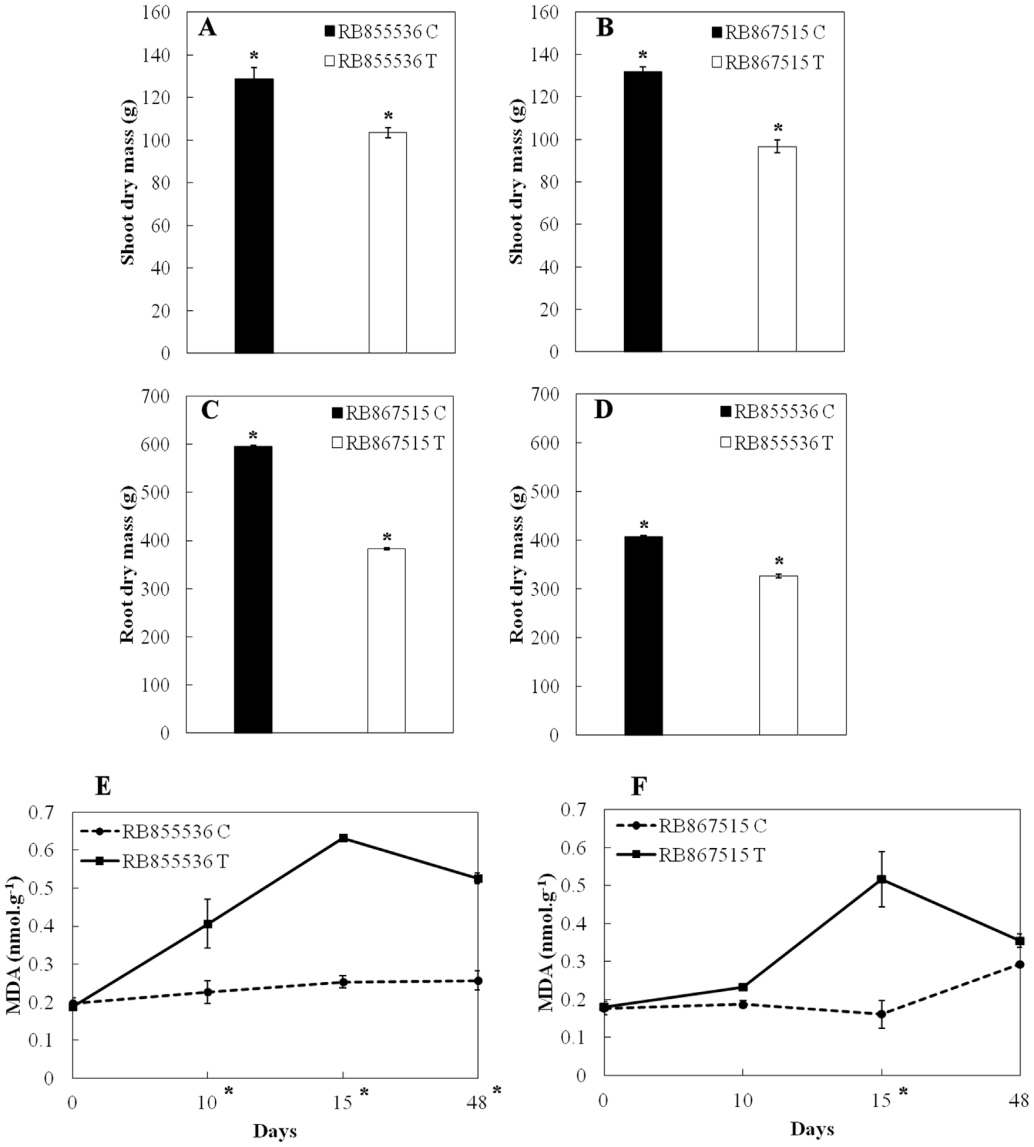
Shoot dry mass for cultivars(A) RB855536 and (B) RB867515; and root dry mass for cultivars (C) RB855536 and (D) RB867515 after being subjected to 48 days of salt stress (100 mM NaCl). Lipid peroxidation levels (MDA) in sugarcane leaves during 48 days of salt stress (100 mM NaCl) for (E) cultivar RB855536 and (F) RB867515. Values are presented as mean ± SD (n = 6 plants). "•" are control plants and "▪" are salt-treated plants. *Significant at p≤0.05.

Salt treated RB855536 and RB867515 plants showed a reduction in shoot dry mass in comparison to control plants (Figure 2A and 2B). Similar results were obtained for roots (Figure 2C and 2D). Comparing the dry mass of controls between the two cultivars, no significant difference was observed for shoots. However, RB867515 control plants showed significantly more root dry mass than RB855536. In relation to malondialdehyde content, cultivar RB855536 plants subjected to salt stress showed a statistically significant increase in lipid peroxidation (MDA) levels from day 10 to 48, with a slight decrease of MDA levels for this last day (Figure 2E). For cultivar RB867515 (Figure 2F), up to day 10, both control and salt-treated plants, showed low values of MDA. However, levels of MDA showed a statistically significant increase in salt-treated plants at day 15 and a decrease at the 48, when MDA levels were similar in control and salt-treated plants. Therefore, MDA levels increased in leaves of salt-stressed plants of both cultivars; however, there was a delay in response for cultivar RB867515 in comparison to cultivar RB855536.

### Macro- and micronutrient leaf concentrations

No significant change in leaf concentrations was observed for any of the macro and micronutrients tested (results not shown), except for manganese. On day 48, there was a statistically significant reduction in Mn^2+^ concentration values in control and salt-treated plants for both cultivars (Figure S1A and S1B in File S1).

### 2-DE analysis of proteins in the sugarcane cultivar RB867515

**Figure 3:**
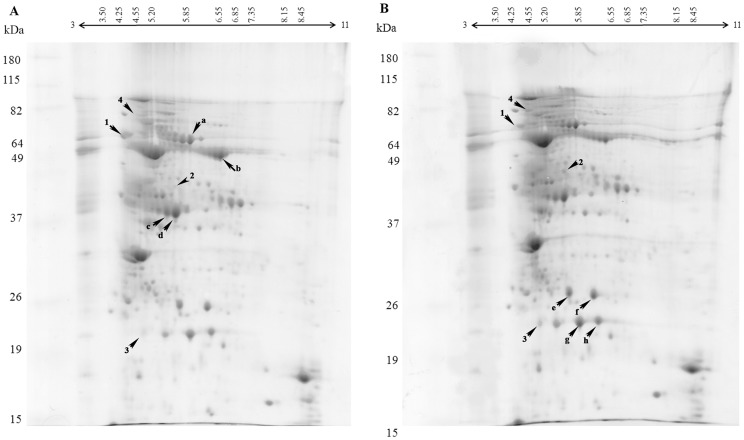
Two-dimensional gel electrophoresis patterns of proteins extracted from sugarcane leaves of the RB867515 cultivar watered (A) with distilled water and (B) after being subjected to 100 mM NaCl for 48 days. The strips used were 13-linear pH gradient of 3-11, stained with Coomassie G-250. The proteins indicated by numbers (1-4) correspond to those showing at least 1.5-fold difference in expression levels between the two different treatments; proteins indicated with letters (a-h) represent proteins with no difference in expression profile.

**Figure 4:**
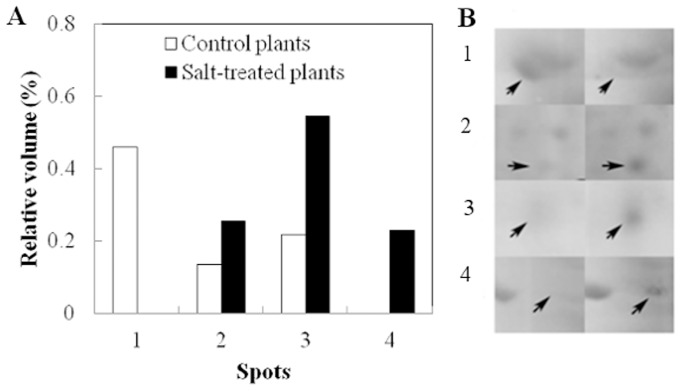
Relative volume of protein spots corresponding to differentially expressed proteins. (A) Quantification of protein expression and (B) Image of protein spots on gels. The left panel shows protein expression on water-treated control plants and right panel shows protein expression on salt-treated plants at day 48. Proteins were identified by mass spectrometry as being (1) fructose 1,6-bisphosphatealdolase; (2) glyceraldehyde-3-phostate dehydrogenase; (3) germin-like protein (4) HSP70. Bars show the mean values of replicate spots.

To identify proteins that are expressed during salt stress in cultivar RB867515, the protein expression profiles of leaves of plants watered with distilled water and with 100 mM of NaCl solution for 48 days were compared using bidimensional protein electrophoresis (Figure 3). Although at days 15 and 48, cultivar RB867515 showed significant changes in some physiological parameters, day 48 was chosen for protein expression analysis due to the greater differences in physiological parameters between water and salt-treated plants, such as a decline in net photosynthesis and leaf water potential (Figure 1). Proteins for both salt-treated and control plants were found mostly in the 4 to 7 pI range. After the second dimension was run, replicates of gels were compared for reproducibility. The gels with highest r^2^ were used to make the reference gels for control (r^2^ = 0.85) and salt-treated plants (r^2^ = 0.84). Comparison of control and salt-treated plants reference gels allowed the identification of proteins that showed at least a 1.5-fold differential expression between gels. Twelve proteins were selected from gels of salt-treated plants and eight proteins were selected from gels of water-treated plants. From a total of twenty selected proteins, twelve were identified (i.e., four showed difference in protein expression and eight were used as control proteins) (Figures S2 to S10 in File S1). Low concentration of proteins in spots precluded identification of the remaining proteins. As shown in Table 1, the four differentially expressed proteins successfully identified were: (1) fructose 1,6-bisphosphate aldolase that was down-regulated in salt-treated plants, (2) germin-like protein that was up-regulated in salt-treated plants and (3) glyceraldehyde 3-phosphate dehydrogenase that was up-regulated in salt-treated plants, and (4) a heat-shock 70 protein that was found only in salt-treated plants (Figure 4A and 4B). Eight additional proteins that showed no change in expression levels were chosen as controls. These were identified as another isoform of fructose 1,6-bisphosphate aldolase, RUBISCO large subunit, ATP synthase CF1 α subunit, 23 kDa polypeptide of PS II oxygen evolving complex and another isoform of germin-like protein.

**Table 1:** Cultivar RB867515 sugarcane leaf proteins differentially expressed between water-treated and salt-treated plants.

Spot #	*Accession* # Gene Index/NCBI	Specie	Protein ID	Sequence coverage (%)	Mowse Score	Theoretical pI/MM (kDa)	Experimental pI/MM (kDa)	Peptide sequences
1	TC113485/ACG36798	*Saccharum officinarum*	Fructose 1,6-bisphosphate aldolase	37.6	67.0	3.50/72.24	5.95/42.014	IVDILVEQGIVPGIK
2	CA117405/ABF93789.1	*S. officinarum*	Glyceraldehyde 3-P-dehydrogenase	22.9	43.0	10.94/54.99	5.33/59.51	TGITADDVNAAFR
3	TC121006/ABG46233	*S. officinarum*	Germin-like protein	20.8	49.0	5.00/22.36	4.60/21.815	VFLFDDAQVKPGVDGLGLSAAR
4	-/CAC16169	*Zea mays*	HSP70	19.6	44.3	4.10/75.78	6.23/23.415	SSVHDVVLVGGSTR
a	-/AAN27981	*Hordeus stenostachys*	RUBISCO large subunit	19.9	31.6	7.00/67.56	6.04/52.766	DTDILAAFR
b	TC112694/YP_054628.1	*S. officinarum*	ATP synthase CF1 α subunit	53.4	43.9	5.89/71.08	6.32/55.294	IAQIPVSEAYLGR
c	-/ACM78035	*Triticum aestivum*	Fructose 1,6-bisphosphate aldolase	26.0	27.4	5.07/53.58	5.48/39.238	AAQDALLLR
d	TC113485/ACM78035	*S. officinarum*	Fructose 1,6-bisphfosphate aldolase	27.8	113.0	5.38/40.00	5.39/40.245	EAAYYQQGAR
e	-/ABY52939.1	*Oryza sativa*	Polypeptide 23 kDa of PS II	16.2	21.0	5.49/26.00	8.28/28.315	QYYSWVLTR
f	CA184061/ABY52939.1	*S. officinarum*	Polypeptide 23 kDa of PS II	16.2	75.0	6.50/26.00	8.66/26.939	TNTDYLAYSGDGFK
g	TC121006/ABG46233	*S. officinarum*	Germin-like protein	15.0	21.0	5.88/22.56	5.96/21.815	PGVDGLGLSAAR
h	TC121006/ABG46233	*S. officinarum*	Germin-like protein	16.3	66.0	6.63/23.20	6.49/21.815	AAVTPAFVGEFPGVDGLGISAAR

Proteins were trypsin digested and identified by MALDI-ToF/ToF MS. “1-4” are differentially expressed proteins and “a-h” are proteins with no difference in protein expression.

## Discussion

### Physiological and biochemical analysis

Many physiological functions in plants are affected by soil salinity and the effects of prolonged stress were observed in sugarcane leaves. Both sugarcane cultivars showed a decrease in their photosynthetic rates during the experiment. In spite of being considered drought tolerant, and the fact that there are similarities between drought and salt-stress responses, RB867515 did not behave as a salt tolerant cultivar in controlled experiments. However, the decrease in photosynthetic rates may also have been a response caused by the decrease of water potential for both cultivars. Stepien and Kłobus [23] working with several concentrations of NaCl in cucumber (*Cucumis sativus* L.) observed a decrease in net photosynthesis due to increasing water deficit. According to Suzuki and Esteves [24], salinity can affect net photosynthesis by changing mesophyll cells' structure and by reducing water availability, thus decreasing the water potential. The presence of salts in the soil solution leads to decreased osmotic potential of the solution, inducing a shortage of water in plants which accounts for the resemblance between drought and salt-stress responses [11].

Decrease in growth of both shoots and roots are a well-known effect of increased salinity. In our experiments, salinity reduced shoots' and roots' mass, affecting both cultivars similarly. Interestingly, however, greater root mass was observed in cultivar RB867515 water-treated control plants in comparison to cultivar RB855536. Since cultivar RB867515 is considered by farmers to be more drought tolerant than cultivar RB855536, the fact that RB867515 had a more developed root system in control plants could help to explain this observation. A deeper root system may lead to drought tolerance as the plant has access to water in deeper layers of soil. In theoretical studies on the potential yield of sugarcane in São Paulo, van der Berg and collaborators [25] observed that the higher the root volume per layer, higher is also the potential yield of the crop for sugarcane plants of first and second cuts. Moreover, the authors also showed that the yield tends to increase with increasing volume of roots. Morris and Tai [26] tested 12 varieties of sugarcane in different water regimes and observed the effect on the development of roots and leaves. The amount of roots in the upper layers was higher in comparison to the lower layers. However, the diameter of the roots was increased in the lower layers. This result is in agreement with those found by Laclau and Laclau [27], where the greatest amount of roots with smaller diameter was found in the upper layers of soil in irrigated culture and the largest amount of roots with greater diameter in the deeper layers in rainfed crops. The maximum depth of sugarcane roots, however, is not a consensus. Smith *et al*. [28] report that root water uptake activity is restricted to a depth of 1.5–2.0 m, but Evans [29] observed this activity at a depth of 6.0 m for sugarcane roots. The size and distribution of the root system of plants is deeply affected by the availability of water in soil, which causes differences in the ability of crops to exploit resources in the lower soil layers [28]. Tolerance of sugarcane to water deficit in places where water is present in deep soil layers may imply an increase in root mass, length and diameter of the root [30]. It is important to note, though, that unlike drought, salt is expected to stress plants continually, from the time of emergence. Although drought tolerance was not tested here and the roots of both cultivars were similarly susceptible to salt, the results obtained suggest that farmer's observations that cultivar RB867515 is more tolerant to drought than RB855536 may be due to its greater root mass.

Reactive oxygen species (ROS) oxidize membranes with the increase of abiotic stresses like salt and drought. Active oxygen species cause deterioration of lipid membranes in plant cells and the levels of peroxidation are measured in terms of MDA content [31]. The RB867515 cultivar resisted to salt-stress conditions until day 15, in contrast to the RB855536 cultivar which showed an increase in MDA levels starting on day 10. However, on day 48, a decrease in MDA levels was observed for both cultivars. This decrease could be due to the presence of detoxification enzymes acting under the ROS [32]. Moradi and Ismail [33] observed that, under different levels of salt stress, rice tolerant to salinity responded to this stress producing lipid peroxidation, however with no statistical differences between tolerant and control plants. Our results were similar to those reported by Shao *et al*. [34]. Working with ten wheat genotypes and several water deficit levels, they were able to separate them according to the production of anti-oxidant enzymes and the production of MDA in each level of stress (mild, moderate and severe). The varieties which showed a greater production of MDA had a lower production of anti-oxidant enzymes. Moreover, in the genotypes that showed an increased production of enzymes, the production of MDA was lower. Although we did not directly test enzyme activity, these results highlight the importance of antioxidant enzyme activity in plants to adverse actions of salt stress, indicating the presence of different pathways to adapt to water stress.

### Proteomic analysis

The study of global patterns of protein expression via various proteomics techniques has gained a lot of attention in recent years. Assessment of mRNA expression has an important caveat which is that multiple layers of regulation of gene expression can lead to situations where mRNA expression levels are not mirrored by protein expression levels [35]. Given that the protein is the active biomolecule in the cell, studying the proteome becomes crucial.

Few proteomic studies have been performed using sugarcane. In studies of sugarcane under the related abiotic stress of drought, Jangpromma *et al*. [36] described an increased expression of an 18 kDa protein. In other report by Jangpromma *et al*. [37], the 18 kDa protein, named p18, was similar to heat-shock proteins or dehydrins and they hypothesized that it may have an important function in protecting the plant against drought, once this protein may help to protect specific cell structures by binding water molecules. Also, p18 may be a stress-inducible heat shock protein, protecting cells from stress injury and helping the folding of new proteins. According to MS/MS, the p18 may be a hydrophilic protein. Hydrophilic proteins are usually charged, which allows them to interact with water or other hydrophilic/polar molecules or to act as molecular chaperones, preventing damaged protein aggregation. Zhou and collaborators [38] verified a change in the expression pattern of proteins in sugarcane leaves submitted to osmotic stress induced by PEG, and reported an increase of two proteins (i.e., 22 kDa protein and RuBisCO small subunit) and the decrease of the other two (i.e., isoflavone reductase-like protein and delta chain of ATP synthase). RuBisCO (ribulose-1,5-bisphosphate carboxylase/oxygenase) catalyses the reaction of D-ribulose 1,5 -bisphosphate and atmospheric CO_2_ to form one molecule of 3-phosphoglycerate and one of phosphoglycolate, being an essential enzyme of the Calvin cycle. The presence of salt in the soil interferes with root water uptake changing the plant water status, increasing leaf water potential and reducing stomatal conductance, therefore reducing photosynthesis. Found in large amounts in the plant leaves, RuBisCO is crucial to provide adequate photosynthetic rates, especially during the salt stress [39]. Ngamhui *et al*. [40] in their work with drought stress and two-dimensional electrophoresis of sugarcane leaf proteins used 13 cm strips ranging from pI 4-7 and identified more than 300 proteins with differences in their expression; and successfully sequenced 19, among them proteins related to photosynthesis, ROS detoxification and defense proteins. The fact they used strips with pI ranging from 4 to 7 may be one of the reasons why these authors identified several proteins that responded to stress. The proteins identified in the present work about salt stress were also in this pI range. The strips used in this study were 13 cm, pI ranging 3-11. Strip selection may have made it difficult to identify proteins that respond to salt stress as they would be compressed in the acidic side of the strip.

A recent study, complementary to this one, addresses changes in sugarcane roots subjected to salt stress [41]. In this study, plants of the same sugarcane varieties used in the present study were cultivated for 45 days and then treated with nutrient solution containing 200 mM NaCl. Samples were harvested for analysis at 2 h and 72 h after treatment. This protocol is in contrast to the one used in the present work where 4 month old plants were treated with water or 100 mM NaCl solution for 48 days.

In this work we have identified four proteins in cultivar RB867515 leaves that respond to salt stress: Fructose 1,6-bisphosphate aldolase (Figure S2 in File S1) was down-regulated, a glyceraldehyde 3-phosphate dehydrogenase and a germin-like protein (Figure S3 and S4 in File S1, respectively) showed increased expression levels under salt stress, and a heat-shock protein 70 (Figure S5 in File S1) was expressed only in salt-treated plants. Our proteome analysis was reproducible, however, the identified changes in protein expression pattern should be confirmed in the future by an alternative technique such as Western blot. The proteins identified are involved in energy metabolism and defense-related responses and their possible participation in helping plants tolerate salt stress is discussed below.

### Proteins involved in energy metabolism

The adaptation of plants to stress is associated with changes in the expressed complement of proteins. It follows that proteomic studies can contribute significantly to the understanding of the relationship between protein abundance and plant acclimation to a stressful environment. Current data indicate more than 2170 identified proteins that respond to stress from 34 plant species, of which 940 or so were identified in leaves of different plants, including the Poaceae family [42], [43], which includes sugarcane. Understanding how the plant responds to stress at a proteomic level, together with data from physiology and biochemistry, can provide directions for how to obtain cultivars with resistance to abiotic stresses such as salinity [44] in sugarcane breeding programs.

Differences in physiological parameters between water-treated and salt-treated plants, such as decrease in photosynthetic rate and water potential, prompted the proteomic analysis of cultivar RB867515 at day 48. The proteins identified for the cultivar RB867515 were involved in energy metabolism processes and are known from studies with other plant species to be early responders of abiotic stresses such as salinity [42], [45]. Fructose-1,6-bisphosphate aldolase (EC 4.1.2.13) is a key enzyme of the energy metabolism, which catalyses the cleavage of β-fructose-1,6-phosphate into D-glyceraldehyde-3-phosphate and dihydroxyacetone phosphate in glycolysis, in addition to the reverse reaction during gluconeogenesis. In this work, it was found that in RB867515 salt-treated plants there was a decrease in the expression of fructose-1,6-bisphosphate aldolase (Figure 4, spot number 1). Sobhanian *et al*. [46] observed similar results when the halophyte grass *Aeluropus lagopoides* was submitted to increased salt levels, some photosynthesis-related proteins, such as fructose 1,6-bisphosphate aldolase, showed decreased expression levels. Different results were obtained by Salekdeh and colleagues [47], who observed that this enzyme increased its expression by 40% in salt-stress rice leaves. Abbasi and Komatsu [48] also observed in the rice leaf sheath an increased expression of FBP aldolase, under different stresses such as cold, salinity and drought, indicating that the plant responded to stressful stimuli by overexpressing this enzyme. There are different reports of a decrease in fructose 1,6-bisphosphate aldolase [49], [50]. According to Chaves *et al*. [51], the expression of some proteins from the Calvin cycle and photorespiration (*e. g*. fructose bisphosphatealdolase) are differently affected by abiotic stresses (e.g., salt and drought) [52]–[54]. These differences may be due to the plant's ability to react differently to imposed stress conditions and the need for growth and compartmentalization of metabolites resulting from the photosynthetic process. Therefore, the response of increased or decreased fructose 1,6-bisphosphate aldolase expression may be due to genetic factors of the plant. It is to be noted though that FBP aldolase was sequenced from different spots on gels (Figure S6 in File S1), indicating that perhaps the presence of isoforms may explain some of the different results in protein expression reported in the literature [45]. Also, FBP aldolase may be an important function in ion vacuole compartmentalization. Barkla *et al*. [55] demonstrate that this enzyme can interact directly with and active the ATPase-depended H^+^ presents in vacuolar membrane, stimulating its ATP binding and hydrolysis activity, important step for salt import into the vacuole, helping the plant cell eliminate the excess of ions Na^+^ and Cl^-^ of the cytoplasm.

Another important enzyme in the energy metabolism is glyceraldehyde-3-phosphate dehydrogenase (EC 1.2.1.12), which was up-regulated in salt-treated plants (Figure 4, spot number 3). This enzyme belongs to the family of dehydrogenases and catalyzes the oxidation of glyceraldehyde 3-phosphate to 1,3-biphosphoglycerate in the glycolytic pathway, in a reaction that produces ATP. This enzyme is also present in the nucleus where it has important roles in gene transcription, DNA replication, DNA repair and RNA export [56]. According to Yang *et al*. [57] overexpression of the glycolysis pathway enzyme is of paramount importance for the increase of soluble sugars accumulation, as well as for providing more energy needed for the plant under stress, and therefore, is an indicator of stress tolerance. The increase in glyceraldehyde-3-phosphatedehydrogenase and the reduction of fructose 1,6-bisphosphate aldolase has been reported for cucumber by Du *et al*. [58], in which these proteins may have altered the activity of the glycolysis pathway, hence the accumulation of soluble sugars would be lower. Sobhanian *et al*. [46] also found a decrease of fructose bisphosphate aldolase in the halophyte grass *Aeluropus lagopoides*, but they also observed an increase in glyceraldehyde-3-phosphate dehydrogenase in response to salinity. The increased expression of this protein may reflect the pattern of carbon flux in response to a reduction in photosynthesis and high demand for the osmotic regulation in the leaves caused by salinity.

### Germin-like protein

Initially described in wheat seeds, germin-like proteins (GLP) have several functions [59], [60] such as receptors and detoxification enzymes [54], [61], [62]. According to Woo *et al*. [63], germin is an apoplastic, glycosylated enzyme with resistance to heat, degradation by proteases and hydrogen peroxide. This resistance may be due to its similarity to desiccation tolerant proteins present in seeds. Germin-like proteins may function as reactive oxygen species (ROS)-scavengers and have a common structure with “true-germin” protein family members, as β-jellyrolls monomers united in a trimer of dimers (homohexamers), with a single manganese ion per monomer. This structure is also similar to other plant ROS-removing enzymes such as Mn-dependent superoxide dismutase (Mn-SOD) [64]. The catalytic processes of these enzymes depend only on the presence of a manganese ion bound between the monomers, with no involvement of other co-factors or specific changes in amino acid residues [63]. Ngamhui and colleagues identified two enzymes involved in ROS detoxification, among them a CuZn-SOD in sugarcane leaves of Thai drought-tolerant cultivars [40].

A GLP with increased protein expression in salt-treated plants was identified in cultivar RB867515 (Figure 4, spot number 2). According to Zimmermman *et al*. [65], germin-like proteins belong to a multigene family – *e.g*. groups of genes from the same organism encoding proteins with similar sequences either in its full length or limited to some specific domain. This would explain the presence of different germin-like proteins with the same molecular mass although with a different pI. The identification of a protein that uses manganese ions during catalysis is interesting because among all macro and micronutrients studied, manganese was the only micronutrient that showed a statistically significant increase in salt-treated plants at 48 days (Figure S1 in File S1). Increased expression of enzymes such as GLP that are responsible for ROS-detoxification together with high levels of manganese could be one of the mechanisms that enables cultivar RB867515 to withstand the adverse conditions of salt stress.

### Defense-related proteins

Molecular chaperones are key components to cellular homeostasis under normal and adverse conditions of growth. They are responsible for protein folding, translocation and degradation processes in normal cell functioning. Most molecular chaperones are stress proteins, many of them identified as heat-shock proteins (HSPs) [66], [67]. Protein spot 4 (Figure 4) present only in salt-stressed plants has been identified as a HSP 70. Aghaei *et al*. [68] investigated the behavior of two contrasting varieties of potatoes (i.e., salt-sensitive and salt-tolerant) in response to 90 mM of salt and found that the overexpression of HSPs occurred only in salt-stress tolerant potato plants. They concluded that HSPs could be considered part of the mechanism that confers salt tolerance in potatoes. In grape, Grimplet *et al*. [69] demonstrated HSP60 expression under drought stress. Studies performed previously by Tiroli and Ramos [70] identified the production of HSP70 in grapes during harvest, which may be considered a stress. After harvest and during ripening, plants undergo a period of dehydration, which can be considered as the main stress factor, initiating the production of proteins responsible for cell turgor and protection against oxidative stress [71] and defense-related proteins, such as heat shock proteins [72]. In sugarcane, there have been reports of the expression of small HSPs (sHSP). Tiroli and Ramos [70] identified a class I sHSP in sugarcane that responded to high temperature stress using ESTs from the sugarcane database. Similar results were obtained by Tiroli-Cepeda and Ramos [19], when they observed that high temperatures induced protein aggregation. Sugarcane plants exposed to high temperatures induced the expression of sHSP class I proteins, which led to increased activity of chaperones in the cell to help previously existing proteins return to function and newly synthesized proteins achieve correct folding. Rodrigues *et al*. [73], despite not having used a proteomic approach, observed an increase in three types of HSP (17.2, 70 and 101) in drought stress tolerant Brazilian sugarcane cultivars. Recently, Ngamhui *et al*. [40] using drought-tolerant Thai sugarcane cultivars described a class IHSP of 16.9 kDa that was up-regulated in sugarcane leaves under drought stress for five days. The expression of HSPs occurred mainly in tolerant plants, a similar result found in this work with RB867515 salt-treated plants, demonstrating that these proteins may participate in the protection of sugarcane against salt stress.

Experiments in which chrysanthemum HSP70 gene was over-expressed in *Arabidopsis thaliana* showed that the increasing in HSP70 expression led to a remarkable tolerance to heat, drought and salinity [74]. Salinity also increases the peroxidation of membranes, and an increase in MDA concentration. The presence of oxidized lipids led to the increase in peroxidase activity. Song and co-workers [74] also noted that membrane damage caused by the action of ROS was lower in plants overexpressing HSP70 compared to the wild type, indicating that the presence of these proteins may be crucial to minimize the damage caused by salinity in plants.

In conclusion, the increase in the glycolytic pathway proteins, such as glyceraldehyde 3-P dehydrogenase, could help the carbon flux through the Calvin cycle leading to an increase in sucrose production [75] and contribute to plant stress tolerance. HSP70 identified in the RB867515 variety, together with GLP (Figure S11 in File S1), may alleviate the damage caused by oxidation, especially in chloroplasts, which might partially contribute to reducing the damage caused by stress, since the decrease in MDA concentrations was observed for day 48 in sugarcane leaves. HSP70 and GLP may protect sugarcane plants against protein unfolding and membrane peroxidation, contributing to the tolerance of sugarcane to moderate salt stress.

## Materials and Methods

### Plant material

Sugarcane cultivars RB855536 and RB867515 were acquired from RIDESA. Culms of both cultivars were grown in vermiculite for a month and then transplanted to 25 cm in diameter pots with drainage holes containing a mix of soil/manure/sand (4∶2∶1, w/w/w). At 4-months, six plants of each cultivar were treated with 100 mM of NaCl solution and other six plants of each cultivar were watered with distilled water (control group) up to field capacity every day in the morning during a period of 48 days. All twenty four plants were randomly arranged.

### Measurements of net photosynthesis and water potential

Net photosynthesis measurements were performed in the morning (8–10 am) after watering. Photosynthesis of the middle third of fully expanded +1 leaves (the first leaf, from top to bottom, with visible sheath, see Figure S12 in File S1) of each repetition was measured using a portable photosynthesis system LICOR-6400 (Li-COR, Lincon, NE, EUA) at 0, 10, 15 and 48 days. After net photosynthesis was measured, +1 leaves were introduced into a Scholander pressure chamber [76]. The applied pressure was increased by increments of 0.2 MPa using nitrogen gas until the xylem sap became visible in the leaf lamina surface. This pressure was considered as the xylem water potential. This analysis was performed at 0, 10, 15 and 48 days of salt stress.

### Determination of dry mass and malondialdehyde content (MDA)

At day 48, shoots and roots of both control and salt-treated cultivars were separated and dried in an oven at 80 °C with forced air circulation and weighted using a digital scale until the mass values became constant. The MDA content of sugarcane leaves was determined according to Hodges *et al*. [77]. Briefly, one hundred milligrams of ground leaves were homogenized with 6.5 mL of 80% ethanol (v/v) and centrifuged for 10 minutes at 16,100 *g*. A total of 1 mL of this extract was transferred to a new tube and 1 mL of 0.65% thiobarbituric acid (TBA) (w/v) in 20% trichloroacetic acid (TCA) (w/v) were added followed by incubation at 95 °C for 25 min. Samples were then transferred to ice for 10 minutes and centrifuged for 10 minutes at 16,100 g. The supernatant was transferred to a new tube and absorbance was read at 532 nm and 600 nm. MDA equivalents in nmol.g FM^−1^ were obtained using the following equation: MDA (nmol.g FM^−1^) = [(A_532_–A_600_)/155000]×10^6^.

### Quantification of leaf nutrient concentrations

For the analysis of macro and micro nutrients, approximately 1 mg of leaf tissue (leaf +1) of each replicate (six plants from controls and six from salt-treated plants) of both cultivars previously pulverized in liquid N_2_ were placed in an oven at 65 °C for 72 hours. Leaf concentrations of P, K, Mg, S, Al, B, Cu, Fe, Mn and Zn were determined in the Analytical Chemistry Laboratory of Embrapa Cerrados (CPAC) by the technique of optical emission spectrometry with inductively coupled argon plasma in a Thermo Jarrell Ash spectrometer model IRIS/AP. Leaf nitrogen concentrations were determined by the colorimetric method described by Kjeldahl [78].

### Protein extraction from leaf material and quantification

At day 48, fully expanded +1 and +2 leaves of six plants (Figure S2 in File S1) from each treatment (control and salt-treated plant) of the RB867515 cultivar were harvested and ground to a fine powder with liquid nitrogen using a mortar and pestle. Total protein extraction was performed according to Wang *et al*. [79]. To remove the photosynthetic pigments, 10 g of powdered leaves were homogenized with 10 mL of 100% chilled acetone, followed by centrifugation at 23,500 g for 5 min at 4 °C. The supernatant was discarded and the procedure was repeated three times. The pellet was then homogenized with 10% trichloroacetic acid (TCA) in chilled acetone (w/v), followed by centrifugation at 23,500 g for 5 min at 4 °C. The supernatant was discarded and this step was repeated three times. The pellet was homogenized in 10% TCA in cold distilled water (w/v), centrifuged (23,500 g for 5 min at 4 °C), and the supernatant discarded, this step was repeated three times. The pellet was resuspended with 80% chilled acetone (v/v), centrifuged (23,500 g for 5 min at 4 °C), and the supernatant again discarded. This step was repeated three times. The final pellet was then homogenized with 10 mL of buffered phenol (Sigma-Aldrich) and 10 mL of solubilization buffer containing 30% sucrose (w/v), 2% SDS (w/v), 0.1 M Tris-HCl buffer (pH 8.0) and 5% β-mercaptoethanol (v/v). This solution was vortex-mixed and centrifuged at 23,500 g, for 5 min at 4 °C. The upper phase (phenolic) was collected, and proteins were precipitated by adding 3 times the volume of a solution containing 0.1 M ammonium acetate in 100% of cold methanol (w/v) overnight at −80°C. Samples were then centrifuged at 23,500 g for 5 min at 4 °C. The pellet obtained was washed twice with 0.1 M ammonium acetate in 100% of cold methanol and twice with chilled 100% acetone. After complete evaporation of acetone at room temperature, the pellet was resuspended in 2% SDS (w/v), 5% glycerol (v/v), 50 mM Tris-HCl buffer (pH 6.8). Protein quantification was performed using the methodology of Lowry *et al*. [80], and the RC DC protein assay kit (BioRad).

### 2-DE and comparative proteome analysis

Seven hundred micrograms of sugarcane leaf proteins (in triplicate for each treatment) were precipitated on ice for 1 h using a 10% TCA (final concentration) solution. After precipitation, proteins were centrifuged at 16,100 g for 20 min at 4 °C. The supernatant was discarded and the pellet washed three times with cold 100% acetone (each wash was followed by centrifugation at 16,100 g for 20 minutes at 4 °C). The pellet was solubilized in a hydration solution (8 M urea, 2% CHAPS, 0.5% IPG buffer with a trace of bromophenol blue and 65 mM DTT) and applied onto a IPG (immobilized pH gel) 13 cm non-linear strip (pH 3-11) (GE Healthcare) by incubation for 16 h at room temperature. The strips were then submitted to isoelectric focusing (IEF) using an Ettan IPGphor 3 (GE Healthcare) apparatus until it accumulated 53,250 v.h^−1^. After IEF, strips were equilibrated in solutions of DTT (50 mM Tris-HCl buffer (pH 8.8), 6 M urea, 30% glycerol, 2% SDS, 1% dithiothreitol-DTT, a trace of bromophenol blue) and iodoacetamide (50 mM Tris-HCl buffer (pH 8.8), 6 M urea, 30% glycerol, 2% SDS, 1% iodoacetamide, a trace of bromophenol blue) for 15 min each, placed on top of 12% polyacrylamide gels according to Laemmli [81] and sealed with agarose solution (25 mM Tris-HCl buffer (pH 8.3), 192 mM glycine, 0.1% SDS, 0.5% agarose and a trace of bromophenol blue). Gels were run until the bromophenol blue reached the end of the gel using the following parameters for electrophoresis: (1) 30 min at 600 V, 90 mA, 100 W and (2) 8 h at 700 V, 240 mA, 100 W. After electrophoresis, gels were fixed in distaining solution (50% distilled water, 40% methanol, 10% acetic acid) for at least 1 h and stained overnight according to Neuhoff *et al*. and Kang *et al*. [82], [83], with Coomassie Blue G-250 (BioRad) (0.1% Coomassie Blue G-250, 2% phosphoric acid, 10% ammonium sulfate and 20% methanol).

### Image acquisition and data analysis

Images of gels were obtained using a scanner HP Scanjet 8290 on photography mode. Analysis of the images was performed using BioNumerics software version 5.10 (Applied Maths, Belgium). Before analysis, all images were converted to gray scale, using the following parameters of the program: TIFF format with OD of 8 bits, 500 kbits, 47% of spot contrast, 75% of spot separation, 25 pixels of spot size and 3 pixels for minimum spot size. For the normalization of gels, one reference gel was created to generate a standard gel, determined by the molecular mass markers (Y), isoelectric point (x) and intensity (z) of spots. After normalization, spots of each gel were connected to the reference gel previously created. Other values remained below the default values of the software. For comparison, the gels had their equivalent spots connected and identified numerically and then the values of volume generated by the program for each spot were used for calculations of correlation. All possible comparisons with the values of volume for each treatment were performed. The gel with the highest correlation coefficient with the other two repetitions was considered representative and was chosen for comparison with the corresponding representative gel from the other treatment (see Figure S13 in File S1). Spot were selected for MALDI-TOF/TOF MS identification using the criteria of 2-fold increased or decreased expression levels.

### In-gel digestion and desalinization of digested proteins

The gel spots selected for identification were excised from the three replicate gels and pooled into 1.5 mL tubes. Protein digestion was performed using the methodology described by Shevchenko *et al*. [84], [85], with modifications. Gel slices were distained overnight and washed with 50% ethanol (v/v) three times, with a 15 min interval between washes. After discarding the ethanol solution, 300 µL of 100% acetonitrile (ACN) was added until gels exhibited a white color. Next, the ACN was removed and 50 µL of 100 mM ammonium bicarbonate and 10 mM DTT were added and tubes were incubated in a water bath at 56 °C for 30 min. After this, the liquid was removed, 50 µL of 100 mM ammonium bicarbonate and 55 mM iodoacetamide were added and tubes were left at room temperature for 90 min. The solution was discarded and the gel slices were washed twice with 100 µL 100 mM ammonium bicarbonate with a 10 min interval between washes. After this, 100% ACN was added until gel slices turned white. Acetonitrile was removed, and tubes were kept at room temperature until the remaining acetonitrile evaporated. Next, tubes were put on ice and 45 µL of 50 mM ammonium bicarbonate containing 5 mM CaCl_2_ and 5 µL of trypsin gold (Promega) were added to each tube for 45 minutes and then incubated at 37 °C for 24 h. The liquid was then transferred to a new tube and dried in a cold speed vacuum. After in gel digestion, proteins were desalted with *PerfectPure* C-18 columns coupled tips (Eppendorf), according to manufacturer's instructions. Desalted proteins were dried in a speed vacuum and solubilized in 10 µL of ultrapure water.

### Identification of proteins through MALDI-TOF/TOF MS, NCBI and Gene index database

Proteins previously digested and desalted were prepared for MALDI-ToF/ToF mass spectrometry analysis using an Ultraflex III instrument (BrukerDaltonics, Billerica, MA). Three microliters of an α-cyan 4-hydroxicynnamic acid saturated solution (1% [w/v] α-cyano-4-hydroxycinnamic acid, 3% [vol/vol] trifluoroacetic acid, and 50% [v/v] acetonitrile) were added to 1 µL of the resuspended sample and applied onto a MALDI target plate in triplicate. Samples were dried at room temperature and the mass spectrometer was operated in reflective mode to obtain the mass spectral profile of peptide fragments generated by trypsin digestion. MS/MS spectra for selected peptides from each protein (around 60 peptides in total) were acquired in LIFT mode. Protein identification proceeded by peptide mass fingerprinting (PMF) and peptide *de novo* sequencing. The peptides masses obtained per protein digestion were compared to the non-redundant plant NCBI database with MASCOT software (MASCOT version 2.2, *Matrix Science*, London) assuming carboxyamidomethylation of cystein and methyonine oxidation as modifications. In parallel, the sequences obtained from the MS/MS spectra were compared to the non-redundant plant NCBI database and Gene Index database (http://compbio.dfci.harvard.edu/tgi/), using organism, max score and max identity as criteria of protein selection.

### Statistical analysis

The photosynthetic rate, water potential and manganese concentration data were analyzed by linear mixed models using individuals as random factors to allow the analysis of repeated measurements over time. The fixed variables of these models were measurement day, treatments, and cultivar type. The measurement day was handled as a categorical variable because of the relatively low number of levels and the lack of a clear linear relationship with the response variables. Instead of using a full factorial model, only the meaningful interactions for this experiment were evaluated. These were day:treatment (the effect of treatment could be different along days), cultivar:treatment (cultivars could respond in different manner to treatments), and cultivar:day (cultivars could have different time dynamics). Since a constant difference among treatments along the entire experiment, including the initial day, was not expected, the main effect treatment was omitted from the models. All models were checked by visual inspection of the residual plots. For some models heteroscedasticity related to day was observed, and under these circumstances variance functions were included in the model. The differences in aerial and root dry mass at the end of the experiment among treated and control experiments were evaluated by t tests. The MDA values were analyzed only by direct observation of descriptive statistics since there were not enough leaves available for biological replicates of treatments and cultivars. All statistical procedures were carried out using the software R version 2.15.2 [86] and the mixed models analysis used also the package nlme [87]. A significance level of 0.05 was used in all tests.

## Supporting Information

File S1 Contains the following files: **Figure S1**. Manganese concentration in sugarcane leaves (mg.kg-1) of cultivar RB855563 (A) and cultivar RB867515 (B) at various timepoints. "•" are control plants and "▪" are salt-treated plants; Values are presented as mean ± SD (n = 6). * Significant at p≤0.05. **Figure S2**. MALDI-ToF/ToF spectrum sequence of fructose 1,6-bisphosphate aldolase (1) of cultivar RB867515 sugarcane leaves treated with 100 mM NaCl for 48 days. **Figure S3**. MALDI-ToF/ToF spectrum sequence of glyceraldehyde 3-P-dehydrogenase (2) of cultivar RB867515 sugarcane leaves treated with 100 mM NaCl for 48 days. **Figure S4**. MALDI-ToF/ToF spectrum sequence of germin-like protein (3) of cultivar RB867515 sugarcane leaves treated with 100 mM NaCl for 48 days. **Figure S5**. MALDI-ToF/ToF spectrum sequence of heat shock protein 70 (HSP 70) (4) of cultivar RB867515 sugarcane leaves treated with 100 mM NaCl for 48 days. **Figure S6**. MALDI-ToF/ToF spectrum sequence of fructose 1,6-bisphosphate aldolase of cultivar RB867515 sugarcane leaves treated with 100 mM NaCl for 48 days. **Figure S7**. MALDI-ToF/ToF spectrum sequence of RUBISCO of cultivar RB867515 sugarcane leaves treated with 100 mM NaCl for 48 days. **Figure S8**. MALDI-ToF/ToF spectrum sequence of ATP synthase subunit α of cultivar RB867515 sugarcane leaves treated with 100 mM NaCl for 48 days. **Figure S9**. MALDI-ToF/ToF spectrum sequence of 23 kDa photosystem II of cultivar RB867515 sugarcane leaves treated with 100 mM NaCl for 48 days. **Figure S10**. MALDI-ToF/ToF spectrum sequence of 23 kDa photosystem II of cultivar RB867515 sugarcane leaves treated with 100 mM NaCl for 48 days. **Figure S11**. Schematic diagram of identified proteins in sugarcane leaves proteome in response to salinity stress. Proteins in stars: up-regulated under saline conditions (100 mM NaCl). Proteins in crosses: expressed only in salt-treated plants under saline conditions (100 mM NaCl). Proteins underlined: down-regulated under saline conditions (100 mM NaCl). Arrows: putative influences on metabolic processes. **Figure S12**. Sugarcane leaves numbering system proposed by Kuijper (1915), with modifications. Leaves +1+2+3 are fully expanded and photosynthetically active. **Figure S13**. Experimental design for comparison and selection of proteins differentially expressed between replicates of control and salt-treated plant gels of sugarcane cultivar RB867515.
(PDF)
