# Supplementary material for: Physiological and Proteomic Analyses of Saccharum spp. Grown under Salt Stress
Source: PLoS One. 2014 Jun 3;9(6):e98463. doi: 10.1371/journal.pone.0098463 (PMC4043529; doi:10.1371/journal.pone.0098463)
Supplement: File S1 — Contains the following files: Figure S1. Manganese concentration in sugarcane leaves (mg.kg-1) of cultivar RB855563 (A) and cultivar RB867515 (B) at various timepoints. "•" are control plants and "▪" are salt-treated plants; Values are presented as mean ± SD (n = 6). * Significant at p≤0.05. Figure S2. MALDI-ToF/ToF spectrum sequence of fructose 1,6-bisphosphate aldolase (1) of cultivar RB867515 sugarcane leaves treated with 100 mM NaCl for 48 days. Figure S3. MALDI-ToF/ToF spectrum sequence of glyceraldehyde 3-P-dehydrogenase (2) of cultivar RB867515 sugarcane leaves treated with 100 mM NaCl for 48 days. Figure S4. MALDI-ToF/ToF spectrum sequence of germin-like protein (3) of cultivar RB867515 sugarcane leaves treated with 100 mM NaCl for 48 days. Figure S5. MALDI-ToF/ToF spectrum sequence of heat shock protein 70 (HSP 70) (4) of cultivar RB867515 sugarcane leaves treated with 100 mM NaCl for 48 days. Figure S6. MALDI-ToF/ToF spectrum sequence of fructose 1,6-bisphosphate aldolase of cultivar RB867515 sugarcane leaves treated with 100 mM NaCl for 48 days. Figure S7. MALDI-ToF/ToF spectrum sequence of RUBISCO of cultivar RB867515 sugarcane leaves treated with 100 mM NaCl for 48 days. Figure S8. MALDI-ToF/ToF spectrum sequence of ATP synthase subunit α of cultivar RB867515 sugarcane leaves treated with 100 mM NaCl for 48 days. Figure S9. MALDI-ToF/ToF spectrum sequence of 23 kDa photosystem II of cultivar RB867515 sugarcane leaves treated with 100 mM NaCl for 48 days. Figure S10. MALDI-ToF/ToF spectrum sequence of 23 kDa photosystem II of cultivar RB867515 sugarcane leaves treated with 100 mM NaCl for 48 days. Figure S11. Schematic diagram of identified proteins in sugarcane leaves proteome in response to salinity stress. Proteins in stars: up-regulated under saline conditions (100 mM NaCl). Proteins in crosses: expressed only in salt-treated plants under saline conditions (100 mM NaCl). Proteins underlined: down-regulated under saline conditions (100 mM NaCl). Arrow [file pone.0098463.s001.pdf]

Figure S1

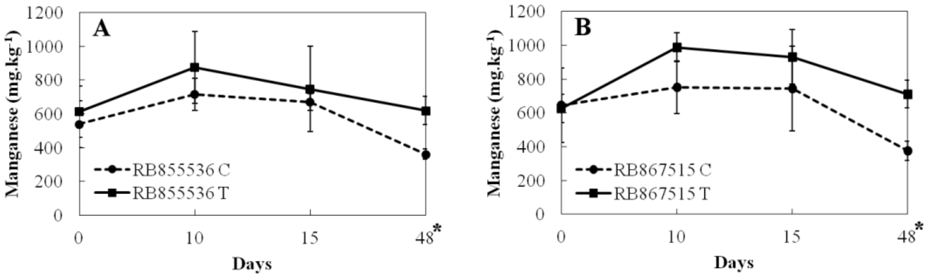
A
Manganese (mg.kg-1)
1200
1000
800
600
400
200
0
RB855536 C
RB855536 T
0
10
15
48*
Days
B
Manganese (mg.kg-1)
1200
1000
800
600
400
200
0
RB867515 C
RB867515 T
0
10
15
48*
Days

Figure S2

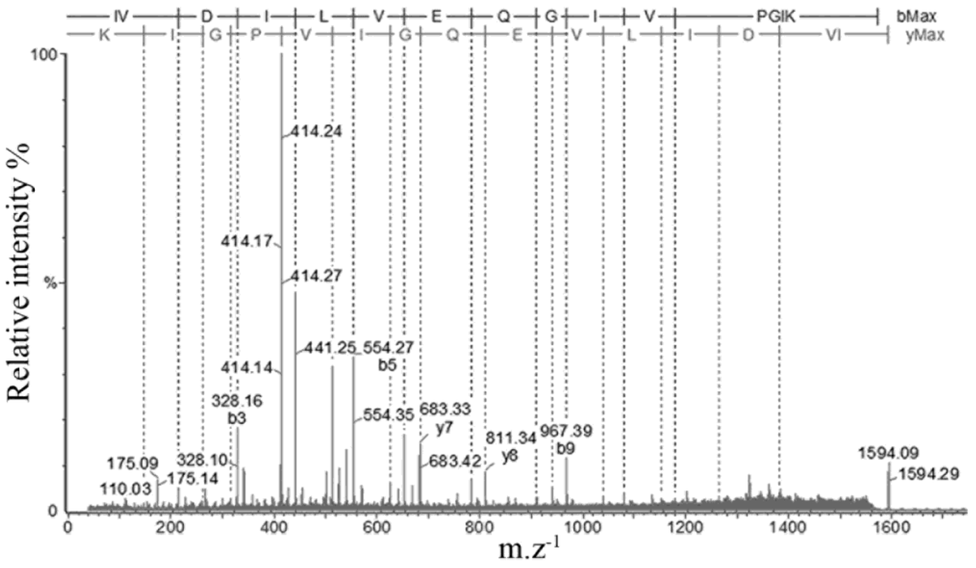
IV
D
I
L
V
E
Q
G
I
V
PGIK
bMax
K
I
G
P
V
I
G
Q
E
V
L
I
D
VI
yMax
100
%
0
Relative intensity %
414.24
414.17
414.27
441.25
554.27
b5
414.14
328.16
b3
554.35
683.33
y7
683.42
811.34
y8
967.39
b9
175.09
328.10
175.14
110.03
1594.09
1594.29
0
200
400
600
800
1000
1200
1400
1600
m.z-1

Figure S3

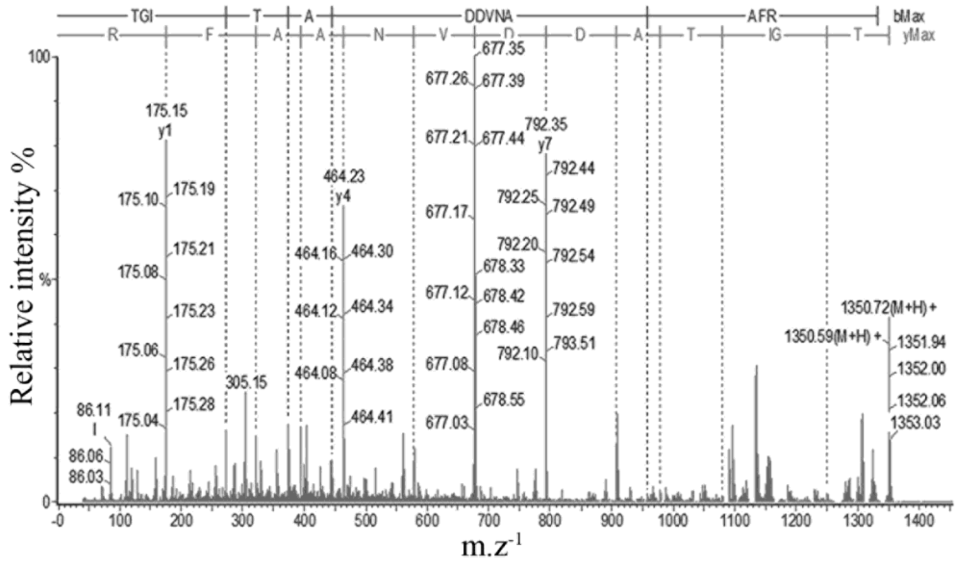
TGI
T
A
DDVNA
AFR
bMax
R
F
A
A
N
V
D
D
A
T
IG
T
yMax
Relative intensity %
m.z-1
175.15
y1
464.23
y4
677.35
792.35
y7
305.15
86.11
1350.72(M+H)+
1350.59(M+H)+

Figure S4

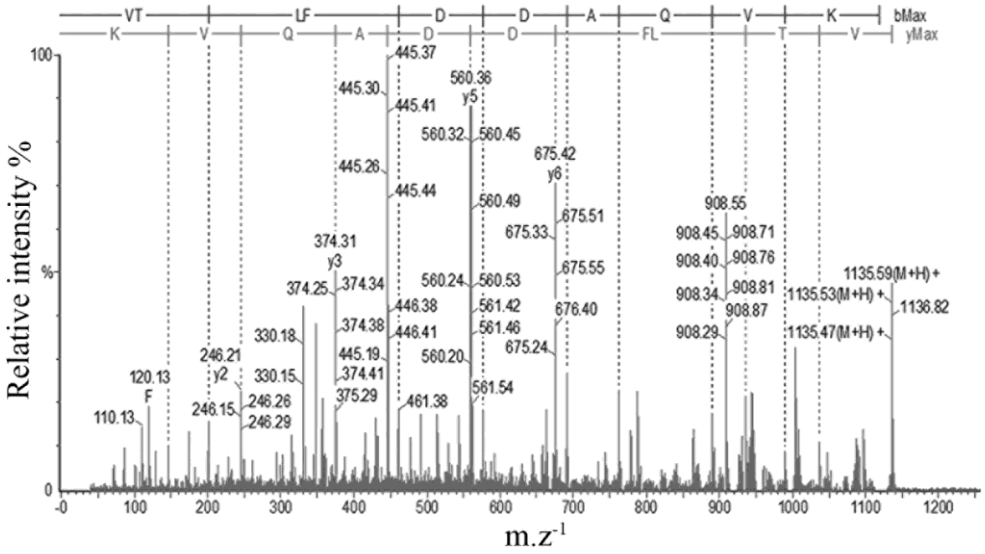
VT
LF
D
D
A
Q
V
K
bMax
K
V
Q
A
D
D
FL
T
V
yMax
100
%
0
445.37
445.30
445.41
560.36
y5
560.32
560.45
445.26
445.44
675.42
y6
560.49
675.51
675.33
908.55
908.45
908.71
908.40
908.76
374.31
y3
374.25
374.34
560.24
560.53
675.55
908.34
908.81
1135.59(M+H)+
1135.53(M+H)+
1136.82
446.38
561.42
676.40
374.38
446.41
561.46
908.29
908.87
1135.47(M+H)+
330.18
675.24
246.21
y2
445.19
560.20
120.13
F
330.15
374.41
561.54
246.26
375.29
461.38
110.13
246.15
246.29
-0
100
200
300
400
500
600
700
800
900
1000
1100
1200
Relative intensity %
m.z-1

Figure S5

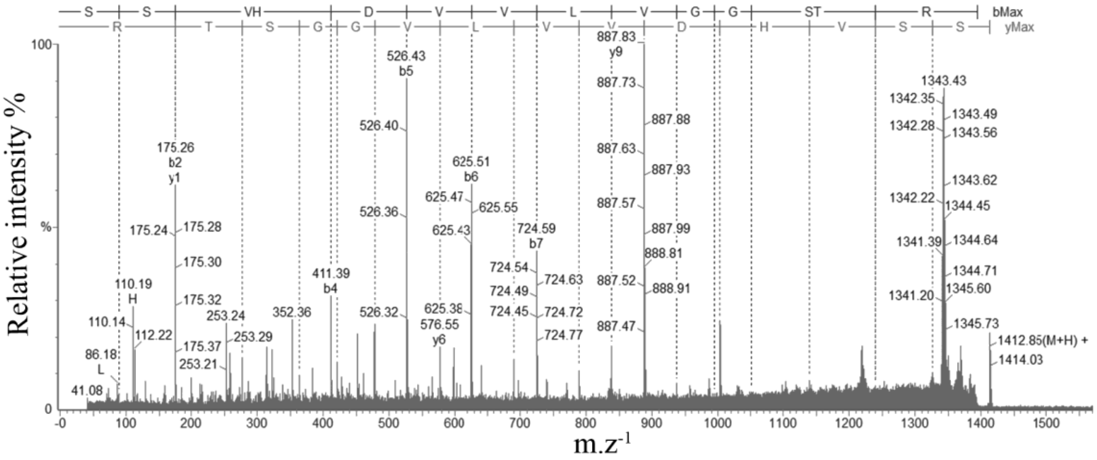

S S VH D V V L V G G ST R bMax
R T S G G V L V V D H V S S yMax
Relative intensity %
100
%
0
526.43
b5
526.40
526.36
526.32
175.26
b2
y1
175.24
175.28
175.30
175.32
175.37
110.19
H
110.14
112.22
86.18
L
41.08
253.24
253.29
253.21
352.36
411.39
b4
625.51
b6
625.47
625.55
625.43
625.38
576.55
y6
724.59
b7
724.54
724.63
724.49
724.45
724.72
724.77
887.83
y9
887.73
887.88
887.63
887.93
887.57
887.99
888.81
887.52
888.91
887.47
1343.43
1342.35
1343.49
1342.28
1343.56
1343.62
1342.22
1344.45
1341.39
1344.64
1344.71
1341.20
1345.60
1345.73
1412.85(M+H) +
1414.03
-0 100 200 300 400 500 600 700 800 900 1000 1100 1200 1300 1400 1500
m.z-1

Figure S6

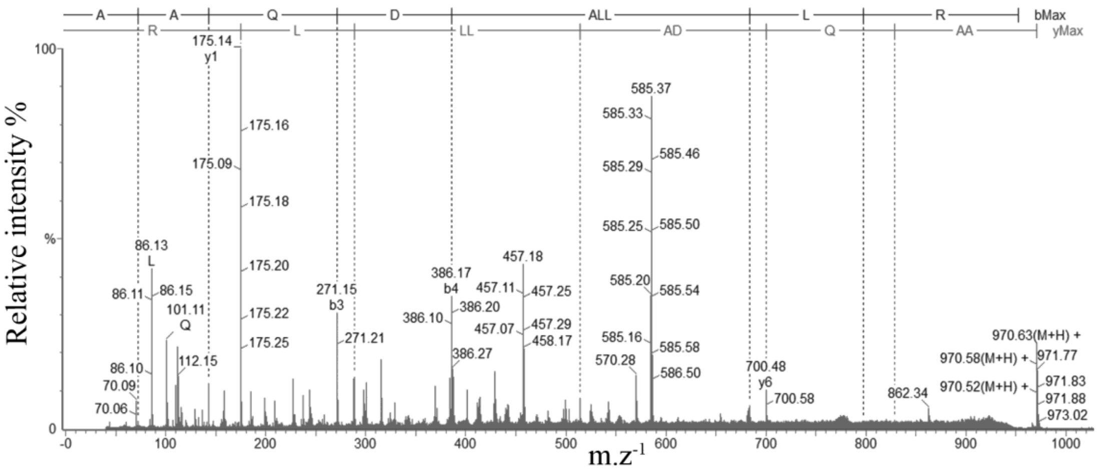

A
A
Q
D
ALL
L
R
bMax
R
L
LL
AD
Q
AA
yMax
Relative intensity %
m.z-1
175.14
y1
585.37
86.13
L
386.17
b4
271.15
b3
457.18
700.48
y6
970.63(M+H) +

Figure S7

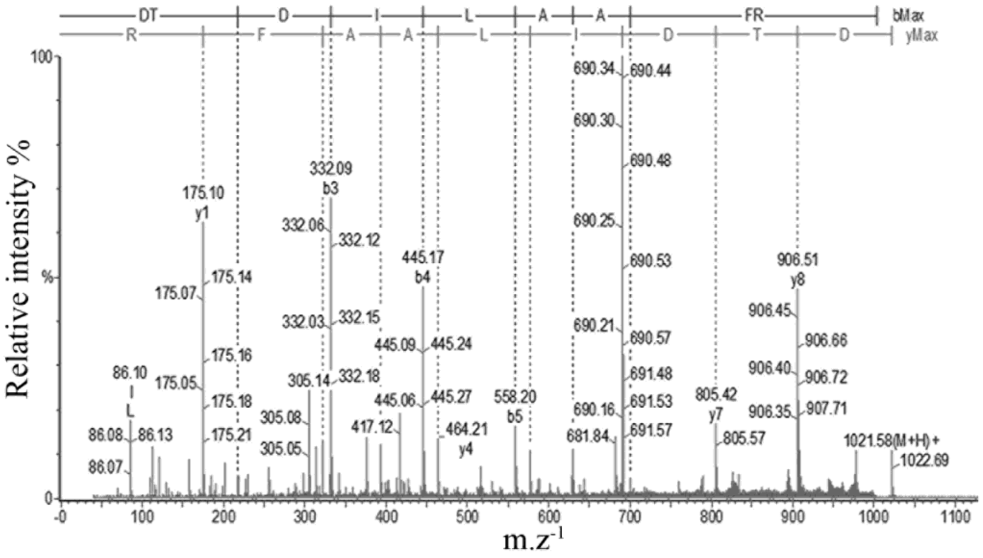

DT
D
I
L
A
A
FR
bMax
R
F
A
A
L
I
D
T
D
yMax
Relative intensity %
m.z-1
175.10 y1
332.09 b3
445.17 b4
558.20 b5
464.21 y4
805.42 y7
906.51 y8
86.10 I L
690.34
690.44
1021.58(M+H)+
1022.69

Figure S8

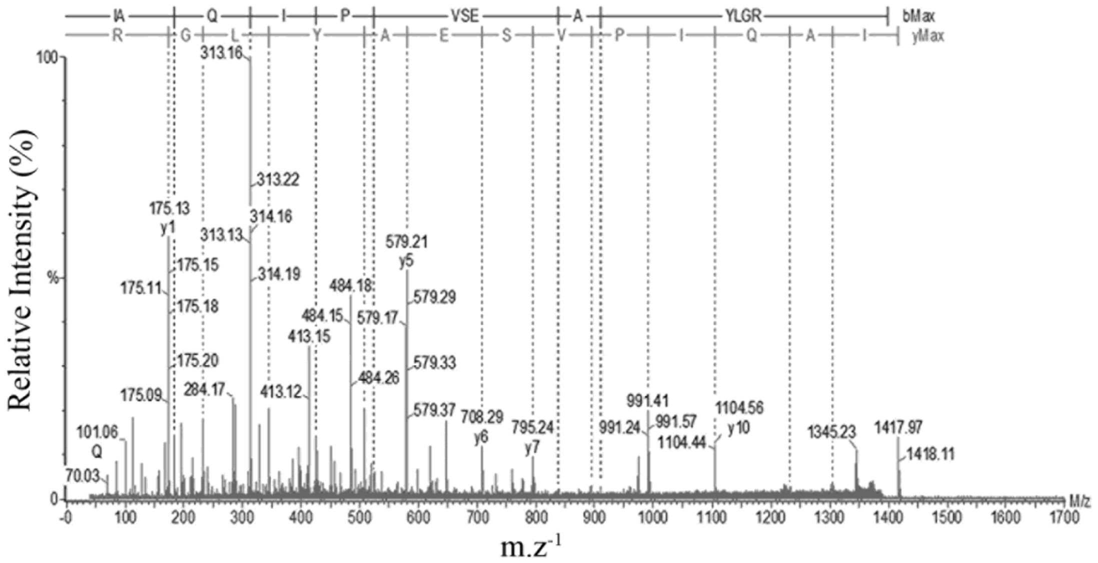
IA
Q
I
P
VSE
A
YLGR
bMax
R
G
L
Y
A
E
S
V
P
I
Q
A
I
yMax
100
%
0
Relative Intensity (%)
313.16
313.22
314.16
313.13
314.19
175.13
y1
175.15
175.11
175.18
175.20
175.09
284.17
101.06
Q
70.03
413.15
413.12
484.18
484.15
484.26
579.21
y5
579.29
579.17
579.33
579.37
708.29
y6
795.24
y7
991.41
991.24
991.57
1104.56
y10
1104.44
1345.23
1417.97
1418.11
M/z
m.z-1

Figure S9

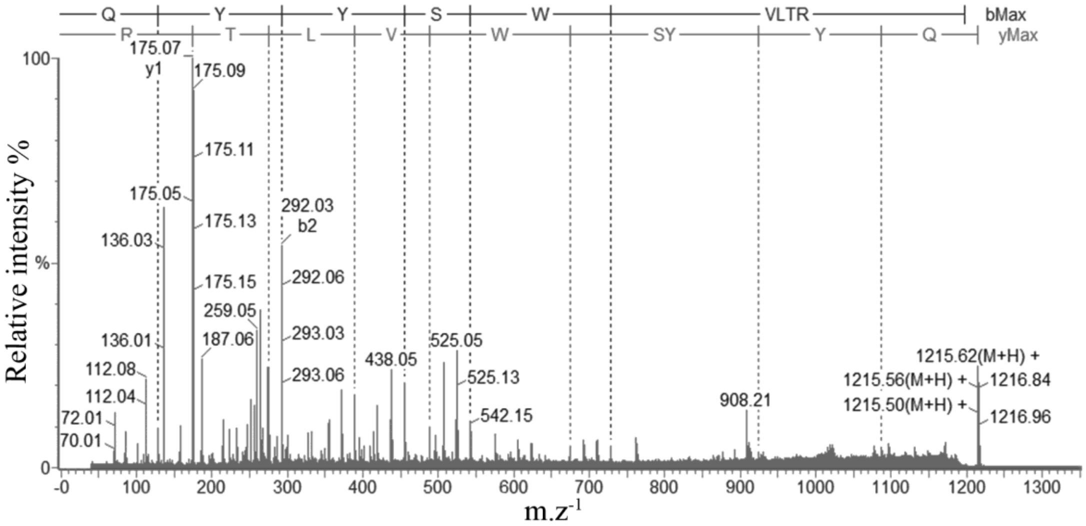

Q
Y
Y
S
W
VLTR
bMax
R
T
L
V
W
SY
Y
Q
yMax
175.07
y1
175.09
175.11
175.05
175.13
136.03
292.03
b2
292.06
175.15
259.05
293.03
136.01
187.06
293.06
438.05
525.05
525.13
112.08
112.04
72.01
70.01
542.15
908.21
1215.62(M+H) +
1215.56(M+H) +
1216.84
1215.50(M+H) +
1216.96
100
%
0
-0
100
200
300
400
500
600
700
800
900
1000
1100
1200
1300
Relative intensity %
m.z-1

Figure S10

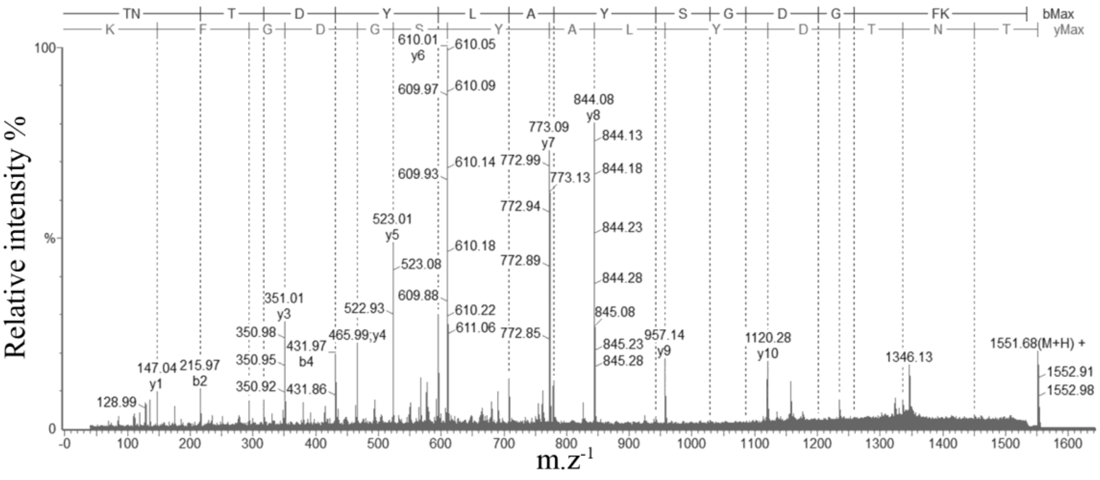
TN T D Y L A Y S G D G FK bMax
K F G D G S Y A L Y D T N T yMax
Relative intensity %
100
%
0
610.01
y6
610.05
609.97
610.09
844.08
y8
844.13
773.09
y7
772.99
610.14
609.93
773.13
844.18
772.94
523.01
y5
844.23
610.18
523.08
772.89
844.28
351.01
y3
609.88
522.93
610.22
845.08
350.98
431.97
b4
465.99;y4
611.06
772.85
845.23
957.14
y9
1120.28
y10
1551.68(M+H) +
350.95
147.04
y1
215.97
b2
845.28
1346.13
1552.91
350.92
431.86
1552.98
128.99
m.z-1

Figure S11

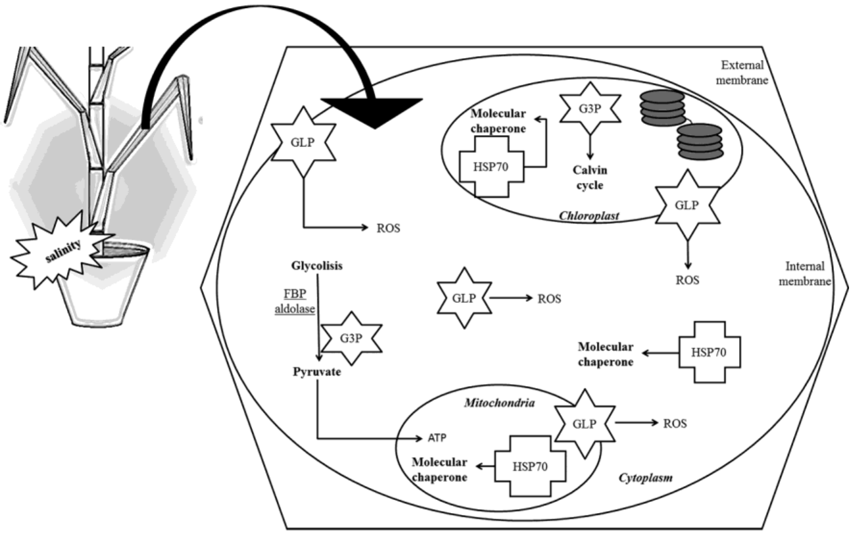
salinity
External membrane
Internal membrane
GLP
ROS
Molecular chaperone
G3P
HSP70
Calvin cycle
Chloroplast
Glycolisis
FBP aldolase
Pyruvate
Mitochondria
ATP
Cytoplasm

Figure S12

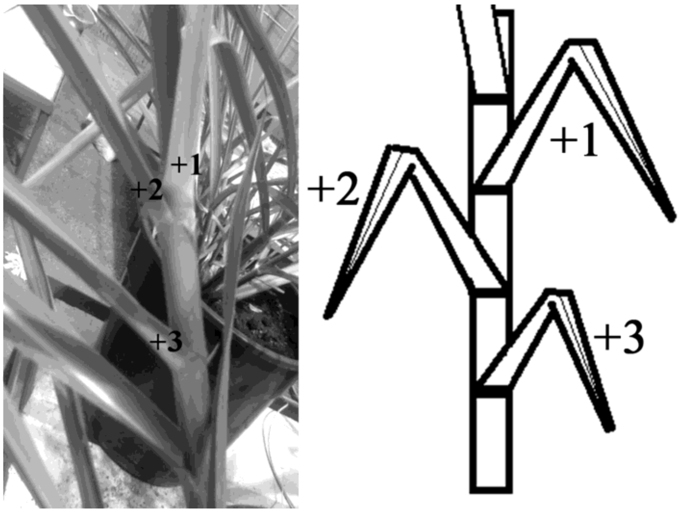

Figure S13

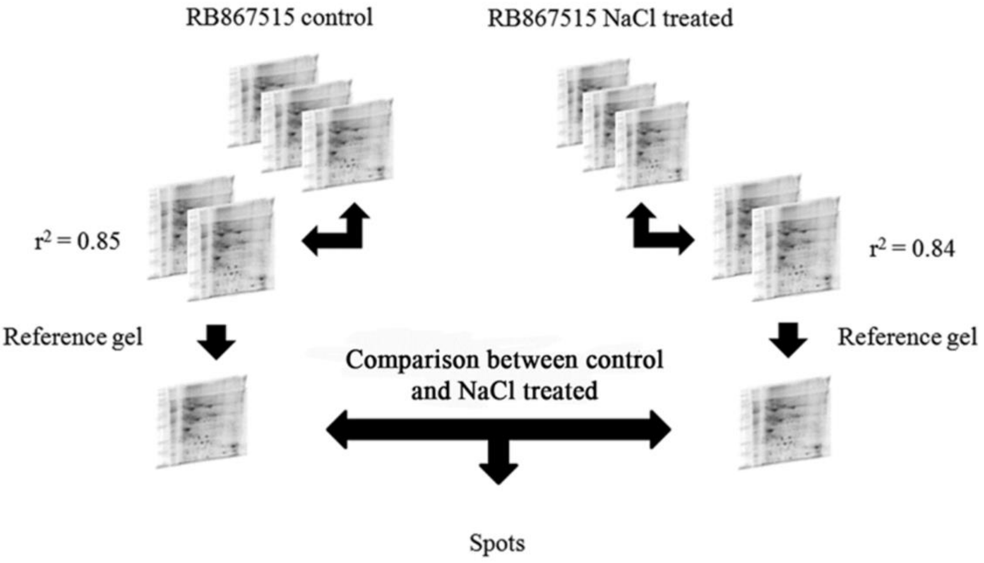

RB867515 control
RB867515 NaCl treated
r2 = 0.85
r2 = 0.84
Reference gel
Reference gel
Comparison between control
and NaCl treated
Spots

Supporting Information Legends

Figure S1. Manganese concentration in sugarcane leaves (mg.kg^{-1}) of cultivar RB855563 (A) and cultivar RB867515 (B) at various timepoints. "●" are control plants and "■" are salt-treated plants; Values are presented as mean ± SD (n = 6). * Significant at $p \leq 0.05$.

Figure S2. MALDI-ToF/ToF spectrum sequence of fructose 1,6-bisphosphate aldolase (1) of cultivar RB867515 sugarcane leaves treated with 100 mM NaCl for 48 days.

Figure S3. MALDI-ToF/ToF spectrum sequence of glyceraldehyde 3-P-dehydrogenase (2) of cultivar RB867515 sugarcane leaves treated with 100 mM NaCl for 48 days.

Figure S4. MALDI-ToF/ToF spectrum sequence of germin-like protein (3) of cultivar RB867515 sugarcane leaves treated with 100 mM NaCl for 48 days.

Figure S5. MALDI-ToF/ToF spectrum sequence of heat shock protein 70 (HSP 70) (4) of cultivar RB867515 sugarcane leaves treated with 100 mM NaCl for 48 days.

Figure S6. MALDI-ToF/ToF spectrum sequence of fructose 1,6-bisphosphate aldolase of cultivar RB867515 sugarcane leaves treated with 100 mM NaCl for 48 days

Figure S7. MALDI-ToF/ToF spectrum sequence of RUBISCO of cultivar RB867515 sugarcane leaves treated with 100 mM NaCl for 48 days.

Figure S8. MALDI-ToF/ToF spectrum sequence of ATP synthase subunit α of cultivar RB867515 sugarcane leaves treated with 100 mM NaCl for 48 days.

Figure S9. MALDI-ToF/ToF spectrum sequence of 23 kDa photosystem II of cultivar RB867515 sugarcane leaves treated with 100 mM NaCl for 48 days.

Figure S10. MALDI-ToF/ToF spectrum sequence of 23 kDa photosystem II of cultivar RB867515 sugarcane leaves treated with 100 mM NaCl for 48 days.

Figure S11. Schematic diagram of identified proteins in sugarcane leaves proteome in response to salinity stress. Proteins in stars: up-regulated under saline conditions (100 mM NaCl). Proteins in crosses: expressed only in salt-treated plants under saline conditions (100 mM NaCl). Proteins underlined: down-regulated under saline conditions (100 mM NaCl). Arrows: putative influences on metabolic processes.

Figure S12. Sugarcane leaves numbering system proposed by Kuijper (1915), with modifications. Leaves +1 +2 +3 are fully expanded and photosynthetically active.

Figure S13. Experimental design for comparison and selection of proteins differentially expressed between replicates of control and salt-treated plant gels of sugarcane cultivar RB867515.
